# Losing Your Sense of Smell: How Bad Is It?—A Comparative Study on the Personal Importance of Smell

**DOI:** 10.3390/brainsci15030218

**Published:** 2025-02-20

**Authors:** Maximiliaan K. P. Becht, Garmt B. Dijksterhuis, Digna M. A. Kamalski

**Affiliations:** 1 Department of Otorhinolaryngology, Head and Neck Surgery, University Medical Centre Utrecht, 3584 CX Utrecht, The Netherlands; 2 Faculty of Psychology and Neuroscience, Section Teaching and Innovation of Learning, Maastricht University, 6229 ER Maastricht, The Netherlands; 3 Brain Centre, University Medical Centre Utrecht, 3584 CX Utrecht, The Netherlands

**Keywords:** olfaction, smell, value, loss of smell, students, psychological, hearing, vision, commodities, gender

## Abstract

The hierarchical perspective on senses has relegated smell to the lowest rank in Western culture while granting vision superiority. Studies show that olfactory impairments, like vision and hearing impairments, reduce quality of life. Our study examines the perceived value of smell in a student population in comparison to hearing and vision, hypothesizing differences based on previous loss of smell (≥2 weeks) and gender. University students were enlisted in a survey comparing smell to vision, hearing, and forfeiting the senses for various commodities (phone, EUR 10,000, hair, and social media). A total of 200 participants completed the survey, with 52 reporting previous loss of smell and 148 reporting no history of smell loss. Overall, smell was the most frequently forfeited sense. While the sacrifice of hearing and vision remained consistent across various commodities, smell was notably forfeited more for certain items. When comparing groups with and without previous loss of smell, no significant differences were observed in forfeiting the senses across various commodities, except for hair. However, it is noteworthy that smell was forfeited more often for all commodities when considering percentages. Furthermore, females exhibited a greater willingness to sacrifice their sense of smell for USD 10,000 and hair. Smell is valued the lowest among the three senses when asked directly and compared to various commodities. There were no significant differences in its perceived value between those with and without previous loss of smell. Furthermore, females tend to value their sense of smell less than males, according to the surveyed commodities.

## 1. Introduction

A hierarchical perspective on the senses has been persisting ever since the time of Aristotle [1]. In Western culture, smell has occupied the lowest rung among the five senses [2], while vision has held a position of superiority [3,4]. This supremacy of vision is believed to have emerged as a result of factors like stereoscopic vision, trichromatic vision, and the expansion of the visual cortex, which may have taken place at the expense of the olfactory capabilities [5,6]. Studies investigating the relationship between quality of life (QoL) and sensory impairments have determined that not only are impairments in vision and hearing linked to decreased QoL, but olfactory impairment also exhibits a negative impact on QoL [7,8,9,10]. The concept of visual dominance is not limited to the neural process alone but also includes the cultural and social supremacy of vision. This is clearly demonstrated in vision-related words dominating the linguistic domain across multiple languages compared to words attributed to the other senses [11]. Nonetheless, it has to be pointed out that in specific non-Western civilizations, such as among the Jahai societies that inhabit the Malay Peninsula’s rainforests, smell assumes significant importance. Within these cultural contexts, individuals find odors as easy to articulate as visual concepts [12]. Our study aims to investigate the perceived importance of the sense of smell in the post-COVID-19 period compared to hearing and vision. To achieve this objective, we designed a short survey based on the prior literature [4], focusing on the valuation of olfaction relative to vision and hearing. By comparing the value of these senses to a few very distinct commodities, such as a smartphone, USD 10,000, hair, or favorite social media platform, we aim to gain insights into the relative, subjective values of these senses. Given the heightened awareness of smell loss resulting from the COVID-19 pandemic, which affected a considerable portion of the student population in the Netherlands, we anticipate increased attention to the negative impact of olfactory impairment. To explore whether this affected the subjective value of olfaction, we recruited individuals from two groups: university students with previous smell loss lasting more than two weeks and those without previous loss of smell. Additionally, we explored whether gender serves as a mediating factor in these findings, considering prior research indicating gender differences in the valuation of smell [4,13,14]. Our hypotheses are as follows: (1) participants would prioritize retaining their sense of hearing and vision over smell; (2) participants with previous smell loss exceeding two weeks would show greater reluctance to sacrifice their sense of smell for a commodity compared to those without previous smell loss; (3) male respondents would exhibit greater resistance to forfeiting their sense of smell than female respondents.

## 2. Materials and Methods

Participants: The recruitment of university students involved the utilization of personal social media platforms such as LinkedIn and Instagram. The survey link was disseminated within WhatsApp groups associated with the first author’s network and expanded through snowballing to other relevant WhatsApp groups. Additionally, a poster outlining the research objectives was strategically placed in diverse locations, including the university and library buildings of the University of Utrecht. Recruitment materials invited students or recent graduates to engage in a concise survey evaluating the significance of the sense of smell in comparison to other senses and commodities, with a prompt to click on a provided link or scan a QR code if interested. Incentives in the form of a chance to win Apple AirPods 2 or gift cards (valued at EUR 50 and 25, respectively) were offered as motivational factors. A formal consent form was used to initiate the survey.

Survey: In January 2024, a comprehensive questionnaire was designed and implemented on Survio.com to explore perceptions of smell, hearing, and vision’s relative value alongside a spectrum of commodities. The survey, originating from Herz and Bajec (2022), was tailored to suit the university student population being investigated. To streamline the survey for the target population, non-essential inquiries such as those regarding income levels and the definition of anosmia were removed. The selection process prioritized retaining questions that had yielded diverse outcomes in the study by Herz and Bajec (2022), therefore excluding several commodities (vacation, pet, shopping, streaming, and little left toe). The iterative development of the survey involved consultations with colleagues to refine draft versions for clarity and relevance. Pilot testing with a small cohort of four individuals provided invaluable insights that contributed to the finalization of the survey. The survey commenced with informed consent followed by demographic inquiries, collecting data on participants’ age, gender, academic status as either a current university student or recent graduate (within one year), and previous sensory impairments in vision, hearing, smell, or none. Participants then faced four key questions, prompting them to make choices between sacrificing their sense of smell, hearing, or vision in exchange for various commodities. The final question probed participants to identify the sense they would least mind losing: sense of smell, hearing, or vision. The comprehensive survey instrument is available in Appendix A.

Procedures: Interested volunteers could access the survey through two avenues: by clicking on the provided link in WhatsApp, LinkedIn, and Instagram posts or by scanning the QR code on posters. Upon entering the survey, volunteers encountered a consent page, agreeing to participate in the study by continuing. Following consent, participants engaged with the survey questions outlined in Appendix A. The survey was designed to be compatible with both smartphones and computers, adapting to the participant’s device automatically. All questions were mandatory except for the email field. Participants were free to discontinue their responses at any moment, and there were no time constraints imposed. On average, volunteers spent approximately 2.2 min completing the survey. In total, 256 volunteers accessed the survey within the designated timeframe, with 214 individuals (83.6%) successfully completing it. Of these completions, 203 were attributed to the direct link provided, while 8 were traced back to the QR code. The survey link and QR code were accessible from 1 February 2024 to 1 March 2024.

Analyses: The analysis involved comparing the university student group with previous loss of smell (US+) to the university student group without previous loss of smell (US−) across all variables. Among the participants, two individuals in the US− group identified their gender as non-binary. Due to the limited number of respondents in this category, data from these two individuals were excluded from the analyses of hypothesis 3 but included in the other analyses. No missing data were reported, as all questions were mandatory to answer, and incomplete survey responses were not stored. In comparing age, gender, and (previous) sensory impairment between the US+ and US− groups, statistical analyses were conducted in IBM SPSS Statistics (Version 29). Gender and sensory impairment were analyzed using the chi-square statistic (χ^2^). To validate the significance of χ^2^ values in settings with a restricted quantity of data, Fisher’s exact test was applied and characterized by ‘f’ [15,16]. To examine the effects of factors (sense modality, commodity, group (loss vs. no loss), and gender) on participants’ preparedness to forfeit a sense, univariate analyses were performed. To quantitatively research preparedness to forfeit a sense for one or more commodities, participants were given one point when choosing a commodity over a sense (smell, hearing, vision) at least once, at any given question. If no commodity was chosen over a sense, zero points were attributed, resulting in a range of zero to maximally one point. All statistical analyses were deemed significant at *p* < 0.05.

## 3. Results

### 3.1. Group Characteristics

A total of 214 respondents successfully completed the survey designed for this research. Among them, 148 individuals comprised the university student group without prior olfactory impairment (US−), while 52 respondents constituted the university student group with prior olfactory impairment (US+). Detailed demographic information is presented in Table 1. A total of 14 participants were excluded due to their non-student or recent graduate status. No difference was observed regarding age distribution between the US− group (M age = 22.1, SD = 2.7) and the US+ group (M age = 21.8, SD = 2.6). Similarly, no gender discrepancy was found between the US− and US+ groups. The US− group comprised 41.9% males and 56.8% females, while the US+ group had 44.2% males and 55.77% females. The majority of respondents in both groups reported no sensory impairment in hearing (US− = 3.4%, US+ = 3.9%) or vision (US− = 1.4%, US+ = 0%).

### 3.2. Hypothesis Testing

#### 3.2.1. Participants Would Prioritize Retaining Their Sense of Hearing and Vision over Retaining Their Sense of Smell

**Figure 1:**
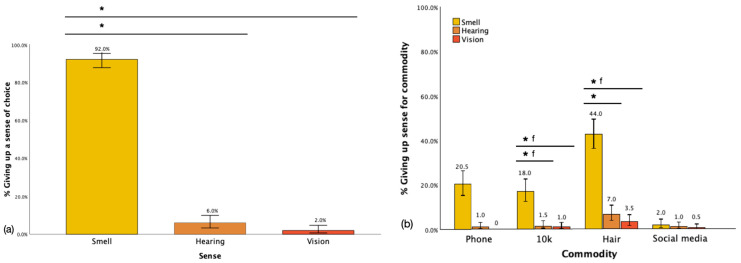
Giving up each sense for all respondents by (**a**) required choice, (**b**) commodities. Note: * indicates statistical significance, ‘f’ indicates use of Fisher’s exact test, error bars show confidence interval of 95%.

Upon being explicitly asked to select a sense to forfeit, a substantial majority (92.0%) of respondents opted to sacrifice their sense of smell. This choice significantly outweighed the preparedness to forfeit either hearing (6.0%) or vision (2.0%) [χ^2^(N = 200) = 146.8, *p* < 0.001 for smell vs. hearing; χ^2^(N = 200) = 46.9, *p* < 0.001 for smell vs. vision], thus confirming our hypothesis indicating the perceived unimportance of smell compared to those other two senses (see Figure 1a). When examining the preparedness to sacrifice the different senses for various commodities, it was consistently found that smell was the preferred sense to forfeit compared to either hearing or vision. Participants displayed a significantly higher inclination to sacrifice their sense of smell when considering hair compared to either hearing (44.0% vs. 7.0%, χ^2^(N = 200) = 7.3, *p* = 0.01) or vision (44.0% vs. 3.5%, χ^2^(N = 200) = 5.1, *p* = 0.05 f). Similarly, when evaluating the value of EUR 10,000, respondents demonstrated a greater readiness to forfeit their sense of smell compared to either hearing (18.0% vs. 1.5%, χ^2^(N = 200) = 13.9, *p* = 0.005 f) or vision (18.0% vs. 1.0%, χ^2^(N = 200) = 9.2, *p* = 0.03 f). No significant difference was observed regarding the sacrifice of smell for their phone (smell = 20% vs. hearing = 1.0%, χ^2^(N = 200) = 0.5, *p* = 1.0 f) vs. vision = 0%, χ^2^ N/A. Notably, when it came to the commodity of social media, smell was overall least frequently forfeited (2.00%), with no significant difference compared to either hearing (1.0%, χ^2^(N = 200) = 0.04, *p* = 1.0 f) or vision (0.5%, χ^2^(N = 200) = 0.02, *p* = 1.0 f). See Figure 1b.

#### 3.2.2. Participants with Previous Smell Loss Exceeding Two Weeks Would Show Greater Reluctance to Sacrifice Their Sense of Smell for a Commodity Compared to Those Without Previous Loss

**Figure 2:**
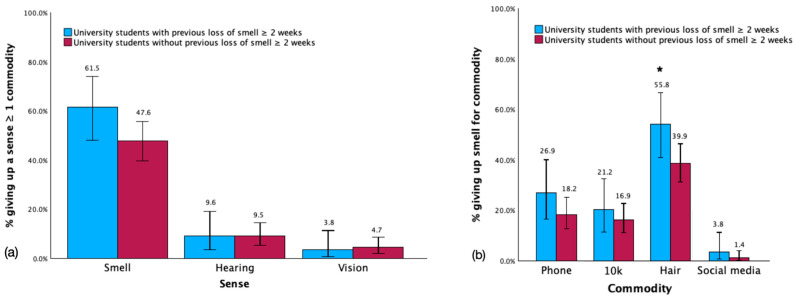
Giving up (**a**) each sense for ≥ one commodity between the university students with and without previous loss of smell for ≥ two weeks, (**b**) smell for each commodity. Note: * indicates statistical significance, error bars show confidence interval of 95%.

In contrast with our hypothesis, a higher percentage of respondents in the US+ group (61.54%) chose to forfeit their sense of smell for at least one commodity compared to those in the US− group (47.30%), although this difference was not statistically significant (χ^2^(N = 200) = 3.12, *p* = 0.11). No significant differences were observed between the US+ and US− groups regarding the preparedness to forfeit hearing (US+ = 9.62%, US− = 9.46%, χ^2^(N = 200) = 0.001, *p* = 1.00 f) or vision (US+ = 3.84%, US− = 4.73%, χ^2^(N = 200) = 0.07, *p* = 1.00 f) for at least one commodity. See Figure 2a. Additionally, further analysis was conducted on the US+ and US− groups to assess their preparedness to sacrifice one of the three senses compared to individual commodities (see Figure 2b). It was observed that a higher percentage of respondents in the US+ group (55.77%) chose to forfeit their ability to smell in comparison to those in the US− group (39.86%) for hair, although this difference was marginally significant (χ^2^(N = 200) = 3.95, *p* = 0.05). No significant differences were observed between the groups concerning their preparedness to forfeit smell in exchange for a phone (χ^2^(N = 200) = 1.78, *p* = 0.23), EUR 10,000 (χ^2^(N = 200) = 0.47, *p* = 0.53), or social media (χ^2^(N = 200) = 1.22, *p* = 0.28 f). See Figure 2b.

#### 3.2.3. Male Participants Would Exhibit Greater Resistance to Forfeiting Their Sense of Smell than Female Respondents

**Figure 3:**
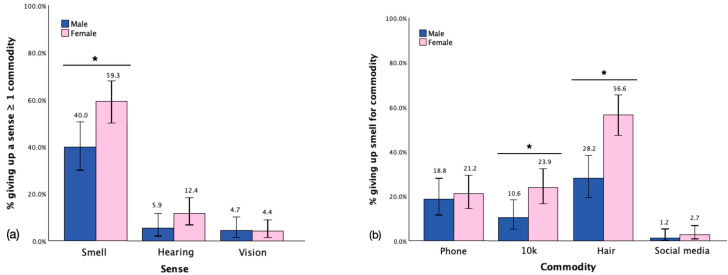
Giving up (**a**) each sense for ≥ one commodity between genders, (**b**) smell for each commodity. Note: * indicates statistical significance, error bars show confidence interval of 95%.

Confirming our hypothesis, the analysis indicated a notable gender difference in the preparedness to sacrifice the sense of smell for various commodities. A higher percentage of females (59.29%) compared to males (40.00%) were prepared to forfeit their sense of smell for at least one commodity (χ^2^(N = 198) = 7.23; *p* = 0.01). No significant differences were observed between genders concerning the preparedness to forfeit hearing (χ^2^(N = 198) = 2.37, *p* = 0.15) or vision (χ^2^(N = 198) = 0.009, *p* = 1.00 f) for at least one commodity. These results are presented in Figure 3a. A more in-depth analysis of the gender effect on the preparedness to sacrifice the sense of smell was conducted by examining the preparedness to forfeit smell for each commodity. The outcomes revealed significant gender differences for hair [females = 56.64% vs. males = 28.24%, χ^2^(N = 198) = 15.85, *p* < 0.001] and EUR 10,000 [females = 23.89% vs. males = 10.59%, χ^2^(N = 198) = 5.77, *p* = 0.03]. However, no significant differences were found between males and females in their preparedness to forfeit smell for a phone (χ^2^(N = 198) = 0.18, *p* = 0.72) or social media (χ^2^(N = 198) = 0.54, *p* = 0.64 f), as illustrated in Figure 3b.

## 4. Discussion

### 4.1. Strengths

In our research, we refrained from disclosing the research objective and underlying hypothesis to individuals close to the researcher. This deliberate choice was aimed at creating a neutral environment and minimizing potential biases stemming from preconceived ideas or anticipations regarding the survey questions. By keeping participants uninformed about the study’s purpose, our aim was to foster objectivity in the responses collected, thereby strengthening the integrity of our data analysis. Our survey methodology drew heavily from the framework established by Herz and Bajec (2022), incorporating identical surveyed commodities (excluding several commodities as mentioned in the section ‘methods-survey’) and question structure. To ensure methodological consistency and enable direct comparisons with the study by Herz and Bajec (2022), we adopted an identical approach to data analysis, employing the same statistical test. This deliberate effort to maintain consistency with the only prior research within the field allowed us to assess the reproducibility of our findings effectively. Through comparative analysis with the sole existing study on this topic [4], our results exhibit substantial coherence, affirming the reliability of our research outcomes.

### 4.2. Limitations

One limitation of our study arises from the sample of respondents. Participants were primarily recruited from Utrecht University or were associated with friends and colleagues of the authors, possibly leading to a limited socio-demographic representation of the Dutch university student population. To address this limitation, it would be imperative to extend the survey to a more diverse student population, including other cities in the Netherlands. However, our findings aligned closely with those of Herz and Bajec (2022) despite their focus on American university students (Boston College, MA, USA and Brown University, Providence, RI, USA). Therefore, our sample may serve as a reliable representation of the broader university student population. Additionally, Utrecht’s central location in the Netherlands, both geographically and in terms of accessibility, positions it as a hub for diverse student demographics. Coupled with its extensive range of academic programs, Utrecht boasts a heterogeneous mix of university students. Furthermore, a reward was offered to incentivize students to complete the survey. The choice to provide a reward stemmed from the challenges associated with gathering respondents from the student population. Unlike patients, who often recognize the significance of contributing to research, students may not have the same level of enthusiasm. However, offering a reward may have introduced participation bias or encouraged respondents with little interest to complete the survey. This possibility may be applicable to respondents who indicated a willingness to forfeit their vision for their favorite social media platform. The number of commodities surveyed was limited due to the voluntary nature of the survey. Considering that students may not always be motivated to participate in surveys, a more time-consuming survey would have resulted in fewer respondents [17,18]. Additionally, as the respondents were collected by one researcher, successful interaction with them resulting in participation was essential to reach a sufficient quantity of respondents. In future research, expanding the scope to multiple cities and universities would enable the inclusion of more commodities with varying psychological and emotional values, providing a broader understanding of the differences between the studied groups. Also, the duration of smell loss was only categorized as either less or more than two weeks without further investigation. It would be intriguing to explore whether the length of this impairment influences the perceived value of smell and to what extent. Additionally, the cause of previous loss of smell was not surveyed. It is conceivable that individuals might assign a lower value to smell if the loss was due to the flu, as they may not fear losing this ability permanently. Conversely, in cases of traumatic brain injury or COVID-19 infection, individuals may fear the prospect of chronic loss of smell, potentially leading to a higher perceived value of this sense. When considering the analyses, the prevalence of olfactory disorders in the general population remains poorly defined. However, it is generally accepted that the incidence of such disorders increases with age [19]. A major limitation in determining prevalence is the lack of a universally accepted diagnostic standard, which complicates cross-study comparisons. Given the uncertainty surrounding prevalence estimates, a formal power analysis could not be conducted in the present study. Consequently, sample size calculations were not feasible, and the decision was made to include as many participants as possible. Lastly, the accuracy of the chi-square test improves with larger sample sizes, whereas small sample sizes can lead to inaccuracies in the chi-square approximation, reducing its reliability [20]. In such cases of sparse data, where the chi-square test may yield misleading results, the Fisher exact test was employed as an alternative.

### 4.3. Hypotheses

**Hypothesis** **1.** *Participants would prioritize retaining their sense of hearing and vision over retaining their sense of smell*.

Our findings highlight the minimal significance attributed to the sense of smell when compared to hearing and vision by respondents. Supporting hypothesis 1, corresponding with the results of previous studies about the topic [3,4]. Respondents displayed a notably greater preparedness to forfeit their sense of smell compared to their hearing or vision. Furthermore, a considerable number of respondents were more inclined to part with their sense of smell to retain various commodities evaluated, surpassing those who were willing to sacrifice their hearing or vision. Hence, despite the challenges posed by the COVID-19 pandemic, olfaction appears to retain its position as the least valued sense. In comparing our findings with those of Herz and Bajec (2022), we observed a higher proportion (92.0% vs. 84.6%) of respondents sacrificing their sense of smell in favor of their ability to hear or see. However, it is important to note that their study included both university students and adults (middle-aged individuals). They observed a higher valuation of adults of their senses (smell, hearing, and vision) when evaluated in the context of various commodities compared to university students. Specifically, the discrepancy in valuation between these demographic groups was notably more pronounced for smell than for hearing and vision. This suggests the possibility that the adult population values their sense of smell more relative to hearing and vision compared to university students. Given that our study focused solely on university students, this discrepancy might account for the higher percentage of respondents ranking smell as the least valuable sense in our study. Further, we found a higher percentage of those surveyed prepared to forfeit their sense of smell in exchange for their hair (44.0% vs. 37.8%). This could be attributed to the above-mentioned reasoning, although one would expect a similar result in the other surveyed commodities (phone, 10 k USD/EUR, and social media). Notably, the distribution of genders differs, with our study comprising fewer females (56.6% vs. 76.9%). This would hypothetically lead to a lower valuation of hair compared to smell. See Hypothesis 3 below for further reasoning. Interestingly, ‘social media’ was found to be the least valued commodity among all those surveyed. For this commodity, respondents were almost equally unwilling to sacrifice their sense of smell as well as their sense of sight or hearing. As stated by Herz and Bajec (2022), the remarkably low importance attributed to social media is particularly unexpected, considering the vast amount of literature discussing society’s fixation and dependency on social media in today’s world. The dynamic nature of popular social media platforms and the constantly changing trends may have diminished the perceived value of the overall concept of social media. Alternatively, this question may reflect a desire to reduce time spent on social media due to its negative effects [21].

**Hypothesis** **2.** *Participants with previous smell loss exceeding two weeks would show greater reluctance to sacrifice their sense of smell for a commodity compared to those without previous loss*.

Although the observed difference was not statistically significant, a larger percentage of respondents from the US+ group (61.5%) were willing to forfeit their sense of smell for at least one commodity compared to the US− group (47.6%), contradicting hypothesis 2. However, the percentages for forfeiting hearing or vision were very similar for both groups, and no significant difference was found across the different commodities. Despite it not reaching significance, this may suggest a differing perception of the value of smell between the groups (US+ and US−). When analyzing the willingness to forfeit the sense of smell across different commodities, a significant difference was found only for the commodity hair. Given that hair was the most retained commodity by far, one would expect the most pronounced differences in this category. Nonetheless, considering the percentages, the US+ groups were more willing to sacrifice smell for all the surveyed commodities (although statistically not significant). Studies examining olfactory dysfunction have consistently indicated a negative impact on QoL. This often manifests in challenges related to cooking, a diminished appetite, and decreased enjoyment of food, as well as difficulties in maintaining personal hygiene and engaging in social interactions [7,8,9,10]. Furthermore, there is evidence suggesting that olfactory impairment is associated with increased depressive symptoms and feelings of loneliness [22]. Notably, in a study conducted by Liljas et al. (2020), a decrease in QoL solely attributable to olfactory impairment was not observed when taste impairment was assessed independently. However, the researchers did recognize the influence of olfactory impairment on flavor perception. When both factors were taken into account, a diminished QoL was evident among the participants in the study. In contrast with previous studies that focused on the QoL of patients experiencing medical complaints attributed to olfactory impairment over extended periods, our study lacks clarity regarding the average duration of olfactory loss, as the cutoff period was established at two weeks. Therefore, comparing our US+ group with the patient groups from previous studies becomes questionable. If such a comparison were to be made, it raises the question of what the potential impact of a diminished QoL on the perceived value of smell would be. When considering a psychological viewpoint, the familiarity effect refers to a cognitive phenomenon wherein individuals exhibit a preference for familiar items over unfamiliar ones. This effect is readily observable in our daily lives, as we often derive comfort from our established routines and lifestyles. Loss of something contributing to this familiarity, whether it is an object or ability, can lead to feelings of discomfort, prompting individuals to assign greater value to the lost entity [23,24]. Over time, however, a new familiarity effect may emerge, influencing perceptions and preferences once again. In our scenario, the short-term impact of the familiarity effect suggests that individuals may initially accord greater importance to the ability to smell until, in the long term, they adapt to the new circumstances. This effect has been described by Baron et al. (2003), considering the QoL of dialysis patients. It was found that while the general public rated the QoL for dialysis patients at 0.39 on a scale from 0 (indicating conditions as severe as death) to 1 (representing perfect health), dialysis patients rated their own QoL higher, at 0.56, thus, showing that patients with chronic health conditions often have a different perspective on their situation compared to those without firsthand experience. Considering the familiarity effect, one might anticipate an increased valuation for the sense of smell when temporarily lost. However, in our study, university students were faced with a choice between sacrificing their sense of smell or a commodity, potentially inducing a similar effect on the perceived value of the commodity. Moreover, respondents were prompted to envision a prolonged loss of smell (depending on their interpretation of the question ‘would you rather lose your sense of smell, or...’ since no timeframe was imposed). Consequently, respondents in the US− group could envision a lower QoL when deprived of the ability to smell (shown by Baron et al. (2003)), thus placing greater value on this sense. Additionally, respondents in the US+ group might not perceive the short-term loss of smell as significantly limiting, as the interpretation of the question regarding previous loss of smell for two weeks or more varies. For some respondents, this loss might signify only a partial dysfunction, which may not significantly impact daily life. Furthermore, a two-week timeframe might be insufficient to experience substantial impairment, resulting in a lesser valuation of its absence. This observation may elucidate why university students who had encountered a loss of smell lasting over two weeks tended to assign a lower value to their sense of smell. Nonetheless, it is essential to note that our study has no insights into the average duration of olfactory loss within the researched population. Additionally, considerable discussion can be had regarding the comparison of QoL with the perceived value of the sense of smell in our study.

**Hypothesis** **3.** *Male participants would exhibit greater resistance to forfeiting their sense of smell than female respondents*.

Despite previous findings indicating heightened olfactory sensitivity and engagement among females, our study did not uncover evidence indicating that females would exhibit a lesser reluctance to forfeit their sense of smell in comparison to males. This outcome aligns with the findings reported by Herz and Bajec (2022) and supports hypothesis 3. The decreased importance attributed to the sense of smell by females in our investigation stemmed from their particularly high valuation of hair and EUR 10,000 compared to males. Women experience hair loss more dramatically than men. As for men with hair loss, women report greater social anxiety, lower self-esteem, and reduced life satisfaction [25]. Hair is usually considered by women as a symbol of their femininity and beauty. Thus, every woman regards any signs of hair loss with great concern. These losses can be deeply internalized, leading to a significant decline in one’s self-esteem and sense of identity, notably when it occurs at a young age [26]. Moreover, societal norms projected on women’s cosmetic appearance compound the negative impact of baldness on their health and wellbeing. The reduced acceptance by society of baldness in women intensifies their distress and, consequently, leads to a deterioration in their overall self-image [24]. Gender differences in attitudes toward money, though complicated, are interesting. Men associate money with power and prestige [27,28], often associated with an increase in attractiveness and desirability [29]. Unlike men, whose view of money is often narrow, women’s perspectives on money are multifaceted. Some studies indicate that women might view money as a source of anxiety and stress [27], which may lead to concerns about its potential adverse effects on relationships [30]. However, other studies identify positive attitudes toward money. Women tend to view money as a security measure [27,31], and they exhibit more financial conscientiousness, such as apprehension about financial management. Emphasizing the importance of balance and financial preparedness [32]. For women, the connection between money and security often serves as a coping mechanism to alleviate anxiety and concerns [31].

In future research, it would be compelling to explore the perceived value of smell among groups experiencing varying durations of smell loss, including those with permanent loss of smell. This could reveal any correlation between the duration of smell loss and its perceived value. Furthermore, broadening the scope of the research to reach a wider pool of potential respondents would enable the use of a larger survey. Consequently, a greater variety of commodities with diverse psychological and emotional significance could be incorporated. This approach would facilitate a more comprehensive understanding of the distinctions between the groups under study.

## 5. Conclusions

In conclusion, our research highlights several key findings regarding the perception of smell in a post-COVID-19 context. Firstly, we reaffirm the prevailing notion that smell remains the least valued sense when compared to hearing and vision. Secondly, while comparing the US− and US+ groups, no statistically significant differences were observed in terms of forfeiting the sense of smell for the surveyed commodities, except for hair. Finally, our results underscore gender-related differences in the valuation of smell relative to the surveyed commodities, with females exhibiting a greater tendency to sacrifice their sense of smell to retain various commodities (hair and EUR 10 k).

## Figures and Tables

**Table 1:** Characteristics of population.

	University Students Without Previous Loss of Smell (US−)	University Students with Previous Loss of Smell (US+)
Total—n; %	148; 74.0%	52; 26.0%
Age—mean (standard deviation (SD))	22 (±3)	22 (±3)
Age, years—range	17–29	18–28
Gender—n; %		
Female	84; 56.8%	29; 55.8%
Male	62; 41.9%	23; 44.2%
Non-binary	2; 1.4%	0; 0%
Prefer not to say	0; 0%	0; 0%
Sensory impairment—n; %		
Vision	2; 1.4%	0; 0%
Hearing	5; 3.4%	2; 3.8%

**Figure A1:**
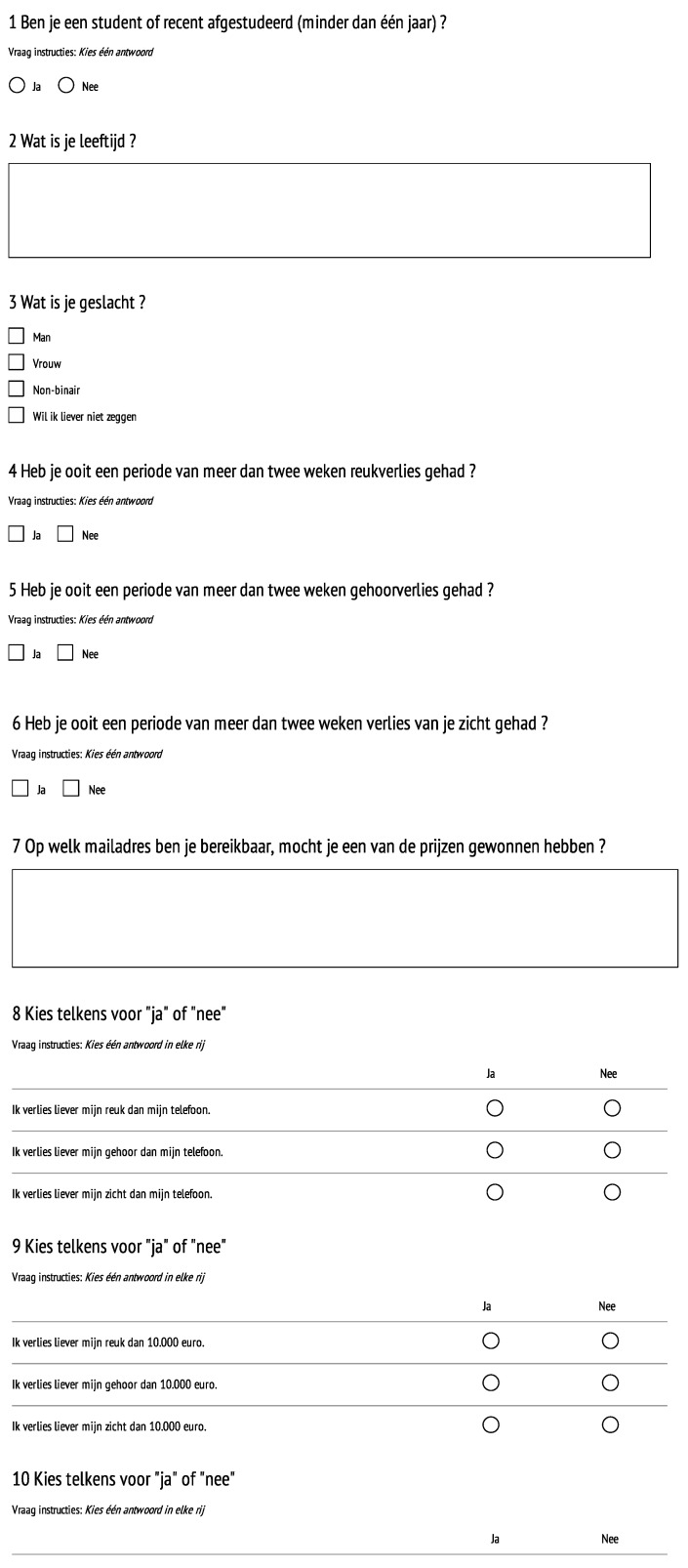

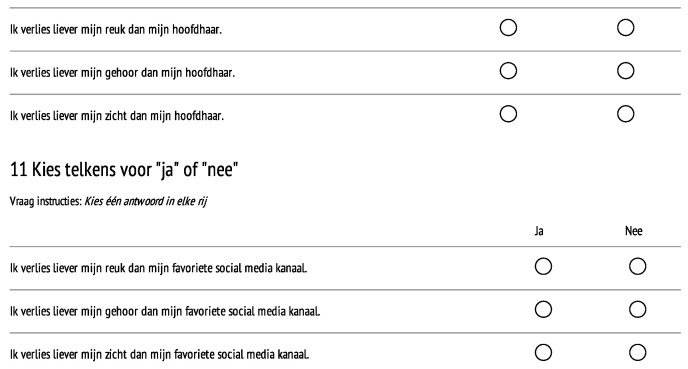
Survey.

**Figure A2:**
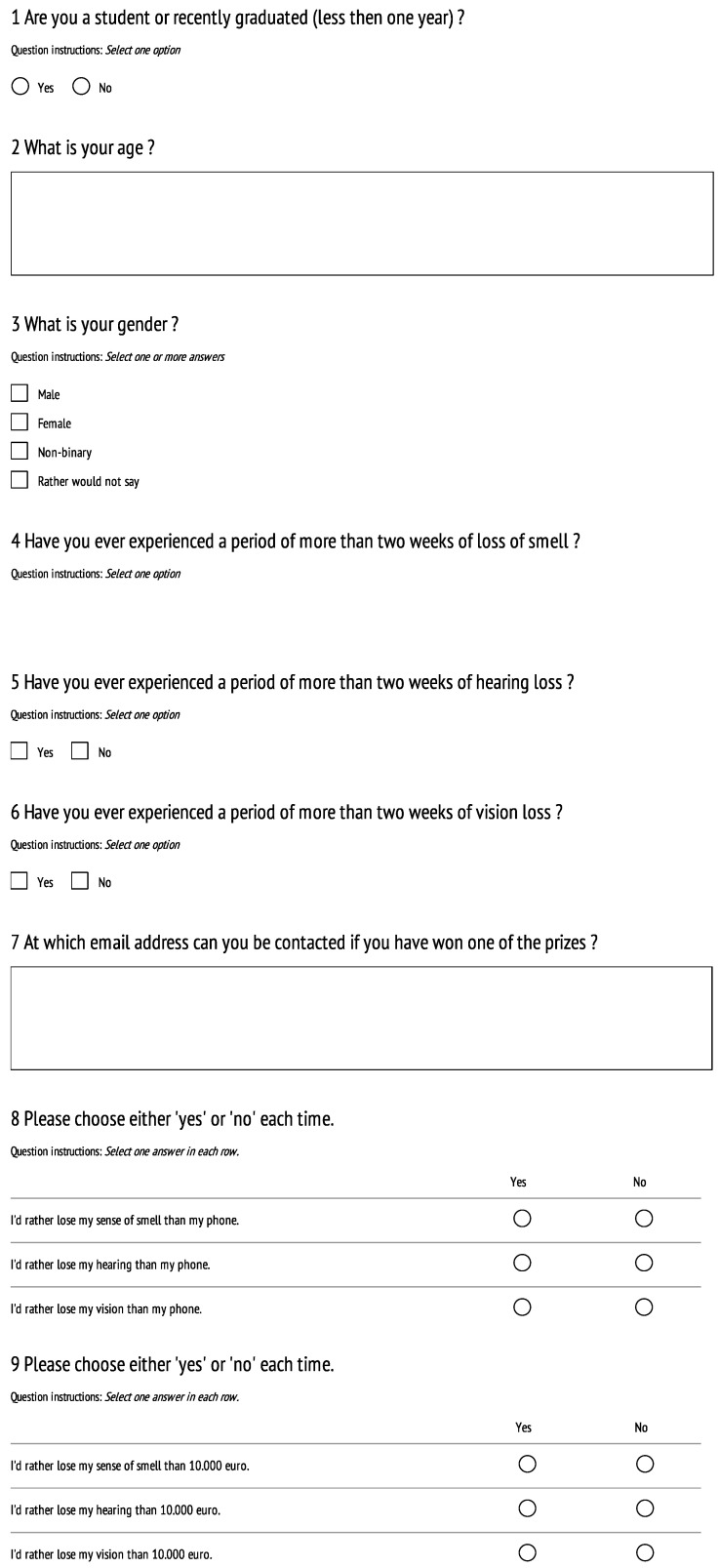

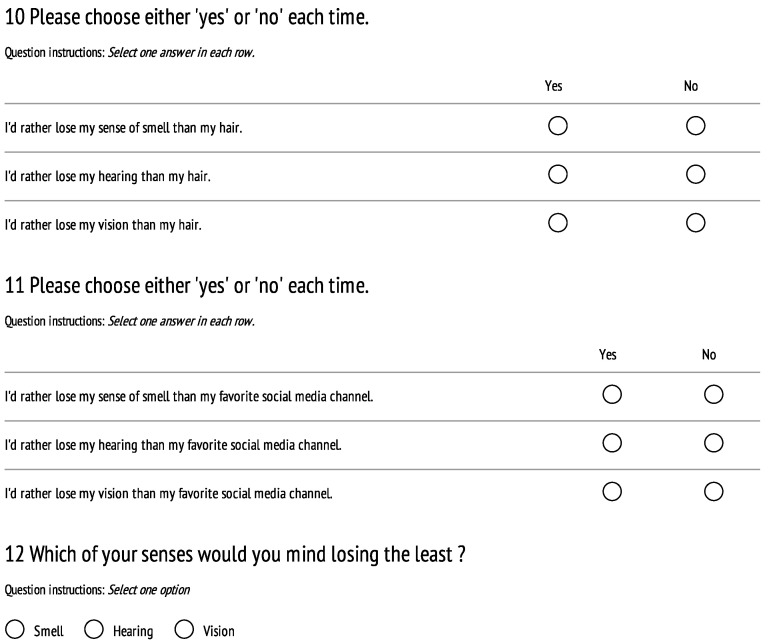
Survey translation.

## Data Availability

The raw data supporting the conclusions of this article will be made available by the authors upon request.

## Institutional Review

Ethical review and approval were waived for this study due to several key considerations. First, the project was structured as a form of social research that utilized participants solely as informants about social conditions in which they were involved, without any physical interaction or practical experimentation. Second, the information collected from respondents was anonymized and did not include any sensitive data, thereby ensuring confidentiality and privacy. Third, informed consent was obtained from all participants prior to their involvement in the survey, clearly outlining the study’s purpose and data usage, including the right to withdraw their responses at any time. These measures collectively ensured that the research adhered to ethical standards while minimizing potential risks to participants.
